# Cocoonase is indispensable for Lepidoptera insects breaking the sealed cocoon

**DOI:** 10.1371/journal.pgen.1009004

**Published:** 2020-09-28

**Authors:** Tingting Gai, Xiaoling Tong, Minjin Han, Chunlin Li, Chunyan Fang, Yunlong Zou, Hai Hu, Hui Xiang, Zhonghuai Xiang, Cheng Lu, Fangyin Dai

**Affiliations:** 1 State Key Laboratory of Silkworm Genome Biology, Key Laboratory of Sericultural Biology and Genetic Breeding, Ministry of Agriculture and Rural Affairs, College of Biotechnology, Southwest University, Chongqing, China; 2 Guangdong Provincial Key Laboratory of Insect Developmental Biology and Applied Technology, Institute of Insect Science and Technology, School of Life Sciences, South China Normal University, Guangzhou, China; University of Michigan, UNITED STATES

## Abstract

Many insects spin cocoons to protect the pupae from unfavorable environments and predators. After emerging from the pupa, the moths must escape from the sealed cocoons. Previous works identified cocoonase as the active enzyme loosening the cocoon to form an escape-hatch. Here, using bioinformatics tools, we show that *cocoonase* is specific to Lepidoptera and that it probably existed before the occurrence of lepidopteran insects spinning cocoons. Despite differences in cocooning behavior, we further show that *cocoonase* evolved by purification selection in Lepidoptera and that the selection is more intense in lepidopteran insects spinning sealed cocoons. Experimentally, we applied gene editing techniques to the silkworm *Bombyx mori*, which spins a dense and sealed cocoon, as a model of lepidopteran insects spinning sealed cocoons. We knocked out *cocoonase* using the CRISPR/Cas9 system. The adults of homozygous knock-out mutants were completely formed and viable but stayed trapped and died naturally in the cocoon. This is the first experimental and phenotypic evidence that cocoonase is the determining factor for breaking the cocoon. This work led to a novel silkworm strain yielding permanently intact cocoons and provides a new strategy for controlling the pests that form cocoons.

## Author summary

Spinning and cocooning are the instincts of many insects, providing a shelter to the residing pupae to resist adverse factors. After the metamorphosis of pupa into adult, the adult must break open the cocoon to emerge, which is called decocooning. We have bioinformatically identified that *cocoonase* is specific to Lepidoptera, and demonstrated that it is the determining factor for breaking the sealed cocoon experimentally for the first time. This work led to a novel silkworm strain yielding permanently intact cocoons and provides a new strategy for controlling the pests that form cocoons and for breeding.

## Introduction

It is well known that insects are the most prosperous animal groups on the earth and distributed in almost every corner of the world [1]. This is benefit to their adaptabilities to the environment during the long-term evolution process, such as the small size conducive to hiding themselves, competitive flight ability, amazing reproduction ability, short life cycle and so on. In addition, insects have astute life-cycle strategies, such as the diapause [2], aposematic signals and mimicry [3], long distance migration [4, 5], etc., which are conducive to the survival and population expansion. The construction of cocoon is one of the effective strategies for some holometabolous insects to protect the immobile pupa from mechanical damage, natural predators, parasites and other adverse factors before adult emergence [6–9].

As known, considerable number of insect species from Coleoptera, Lepidoptera, Hymenoptera and Neuroptera [10–14] were able to spinning and cocooning. The mature insect larva usually use silk or silk embedded with surrounding materials to construct cocoons, such as the silk cocoon of silkworm *Bombyx mori* [11] and mixed cocoon of *Cnidocampa flavescens* Walker [15]. After the pupa metamorphosing into adult, how the adult breaks out of the cocoon to complete the generational development becomes a matter. To the best of our knowledge, insect adults escape from the cocoon through the following way: reserving an emergence valve or weak places on the cocoon [16], utilizing special structure of the head to break the cocoon [17], or secreting alkaline fluid that can soften the cocoon [18, 19].

Previous study has reported that the silkmoth-vomiting fluid contained a kind of protease which could specifically hydrolyzed sericin of the cocoon layer, making the cocoon soft and allowing the moth to escape [20]. In the researches on *Antheraea* moths, it was found that the protease has hydrolytic activity on a variety of proteins, including sericin, denatured hemoglobin, gelatin, etc., and has specific hydrolytic activity on esters, showing the characteristics of trypsin [21, 22]. The protease thereupon was named cocoonase because of its function in hydrolyzing the cocoon. The coding sequence of the *cocoonase* gene was deciphered gradually [23], and subsequent researches have been devoted to analyzing the properties [24–26] and secretory organs [27–30] of cocoonase enzyme and exploring its application value in degumming [31–35]. However, few researches were regarding the origin, evolution and role of cocoonase.

It is particularly noteworthy that, a recent study identified 29 proteins in the silkmoth-vomiting fluid through LC-MS/MS assay [36]. The result implied that some other proteins besides cocoonase in the vomiting fluid may also participate in the cocoon digesting process. To explore whether cocoonase is the unique component enough to dissolve cocoon or other proteins in the silkmoth-vomiting fluid have the same hydrolytic ability, and investigate the origin and evolution of cocoonase, we identified the *cocoonase* genes in a wide range of species and performed evolutionary analysis. Further, we knocked out the *cocoonase* (*BmCoc*) gene in the domesticated silkworms which spin sealed and dense cocoons by CRISPR/Cas9 system to explore its role in the decocooning process.

## Results

### The *cocoonase* is specific to Lepidoptera

**Fig 1:**
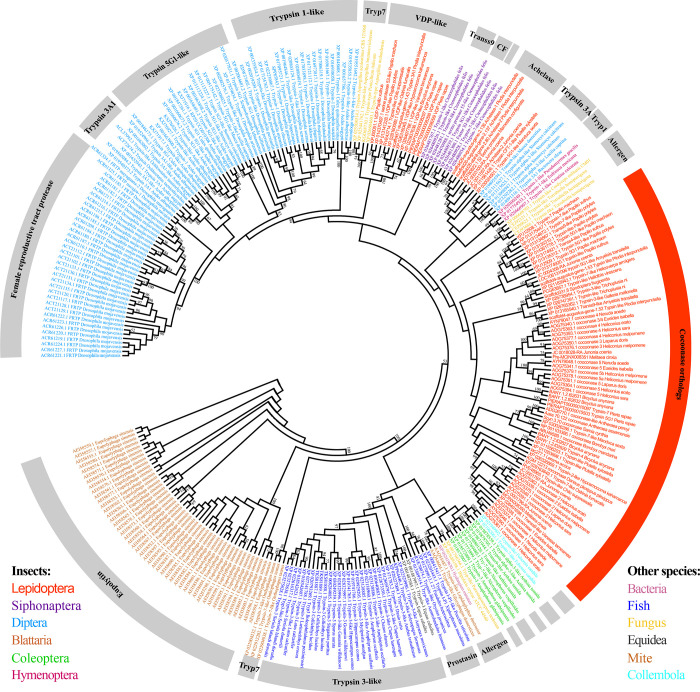
Phylogenetic analysis of cocoonase homologs. The phylogenetic tree was constructed using the Maximum Likelihood method, based on the Poisson correction model with 1,000 bootstrap replicates, and bootstrap support values more than 50% were shown in the tree. The 295 cocoonase homologs from 12 classes, identified with different colored fonts, are divided into 14 clades, showed in the outer ring. And 70 protein sequences from the Lepidoptera were aggregated with the clade containing silkworm cocoonase, shown in the orange curved area.

In order to reveal whether cocoonase is widespread and pervasive among insects, we investigated the distribution of the gene in various species. We used the silkworm cocoonase protein sequence as query object and obtained 295 homologs of cocoonase, all belonging to the serine protease superfamily but with diverse functions (Fig 1). Based on the phylogenetic analyses and protein characteristics, 70 homologs were identified as cocoonase (Fig 1). It is worth emphasizing that these 70 *cocoonase* genes were found within moth and butterfly families, which indicates that the *cocoonase* is Lepidoptera-specific (Fig 1). In addition, multiple copies of *cocoonase* were identified in the butterflies, consistent with recent research on *Heliconius* butterflies, which uncovered the role of duplicated *cocoonase* genes in pollen feeding [37–39]. While, there was single copy in most moths except *Plutella xylostella*, *Trichoplusia ni*, *Plodia interpunctella* and *Amelois transitella*, which had two copies (S1 Fig). The results indicated that the *cocoonase* experienced gene duplication events during the evolution of lepidopterans that resulted in diverse copy numbers and functional divergence.

**Fig 2:**
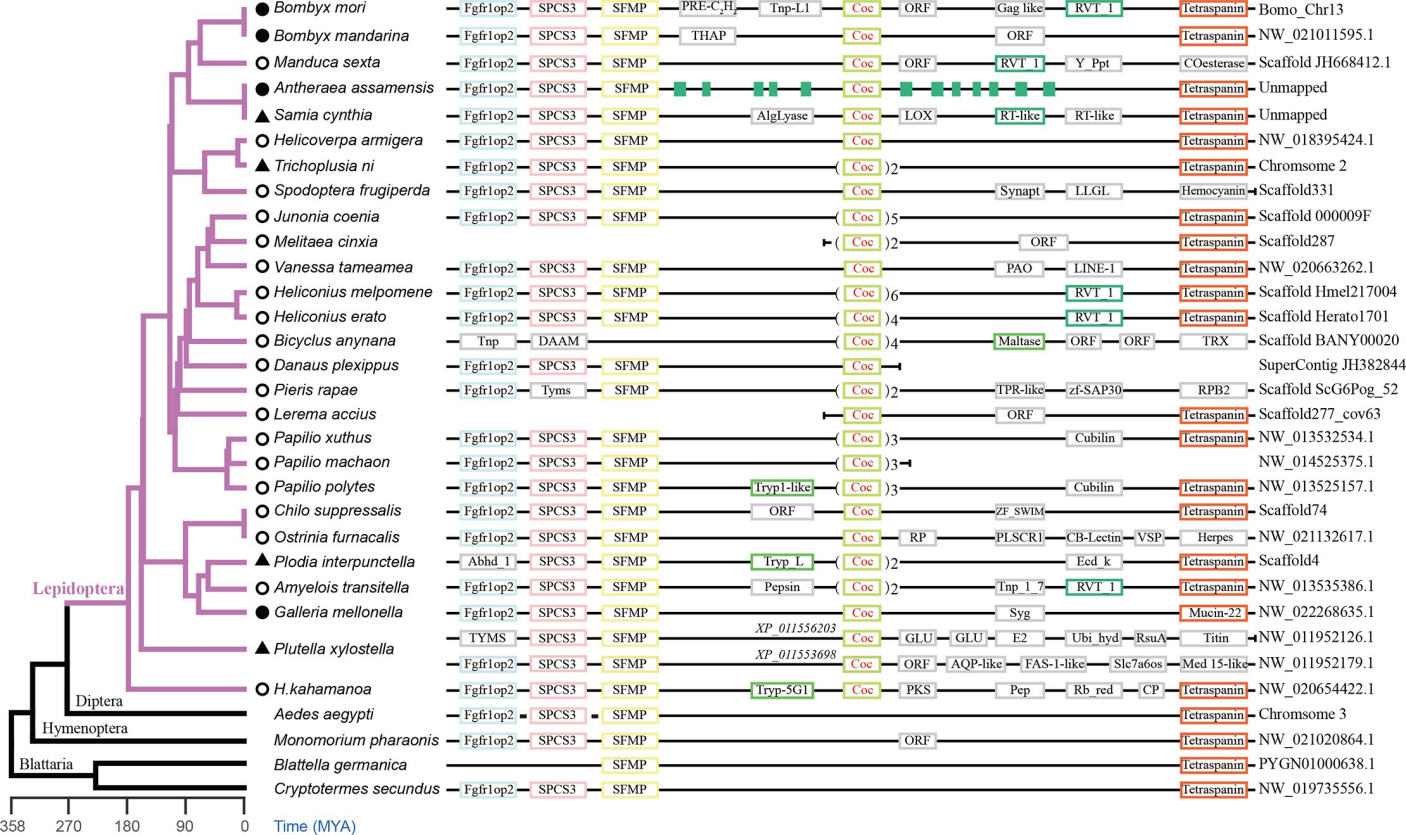
Syntenic analysis of *cocoonase* genes in insects. Syntenic analysis of *cocoonase* genes in lepidopteran species marked with purple clades and three outgroups with available linkage information. Orthologous genes are identified by a border with same color, and non-homologous genes and genes without functional annotation are indicated by ORFs and marked by a gray border. The green rectangles represent predicted transposons. The Arabic numerals in the figure showed the copy number of the *cocoonase* gene in each species. The two *cocoonase* genes of *Plutella xylostella* were located at different scaffold, and the IDs of the two enzymes were shown next to the gene on the scaffold diagram.

The synteny analysis of the *cocoonase* loci showed a conserved gene organization and confirmed the identification of cocoonase. It also indicated that there were abundant gene duplications, physical movements, and loss events within this family. In most lepidopterans, the FGFR1 oncogene partner 2 (*Fgfr1op2*), signal peptidase complex subunit 3-like (*SPCS3*), putative sperm flagellar membrane protein (*SFMP*), *cocoonase* and *Tetrospanin* were arranged in linear order in the genome, except in *Manduca sexta*, *Spodoptera frugiperda*, *Ostrinia furnacalis* and *Pieris rapae* which displayed gene synteny on only one side (Fig 2). Although the two *cocoonase* genes of *Plutella xylostella* were not on the same scaffold, both the two copies showed collinearity in the upstream of *cocoonase* gene. It is worth noting that *FGFR1*, *SPC22*, *SFMP* and *Tetrospanin* are conserved and arranged in linear order in the genomes of other insects, for example, in *Aedes aegypti* (Diptera), *Monomorium pharaonis* (Hymenoptera) and *Blattella germanica* (Blattaria), except that there is no *cocoonase* gene between *SFMP* and *Tetrospanin*. The above results imply that *cocoonase* was obtained from a Lepidoptera ancestor and that gene replication and neofunctionalization occurred following differentiation of species developing in different requirements with diverse living environments.

### Enhanced purifying selection on the *cocoonase* gene in insects spinning sealed cocoons

**Fig 3:**
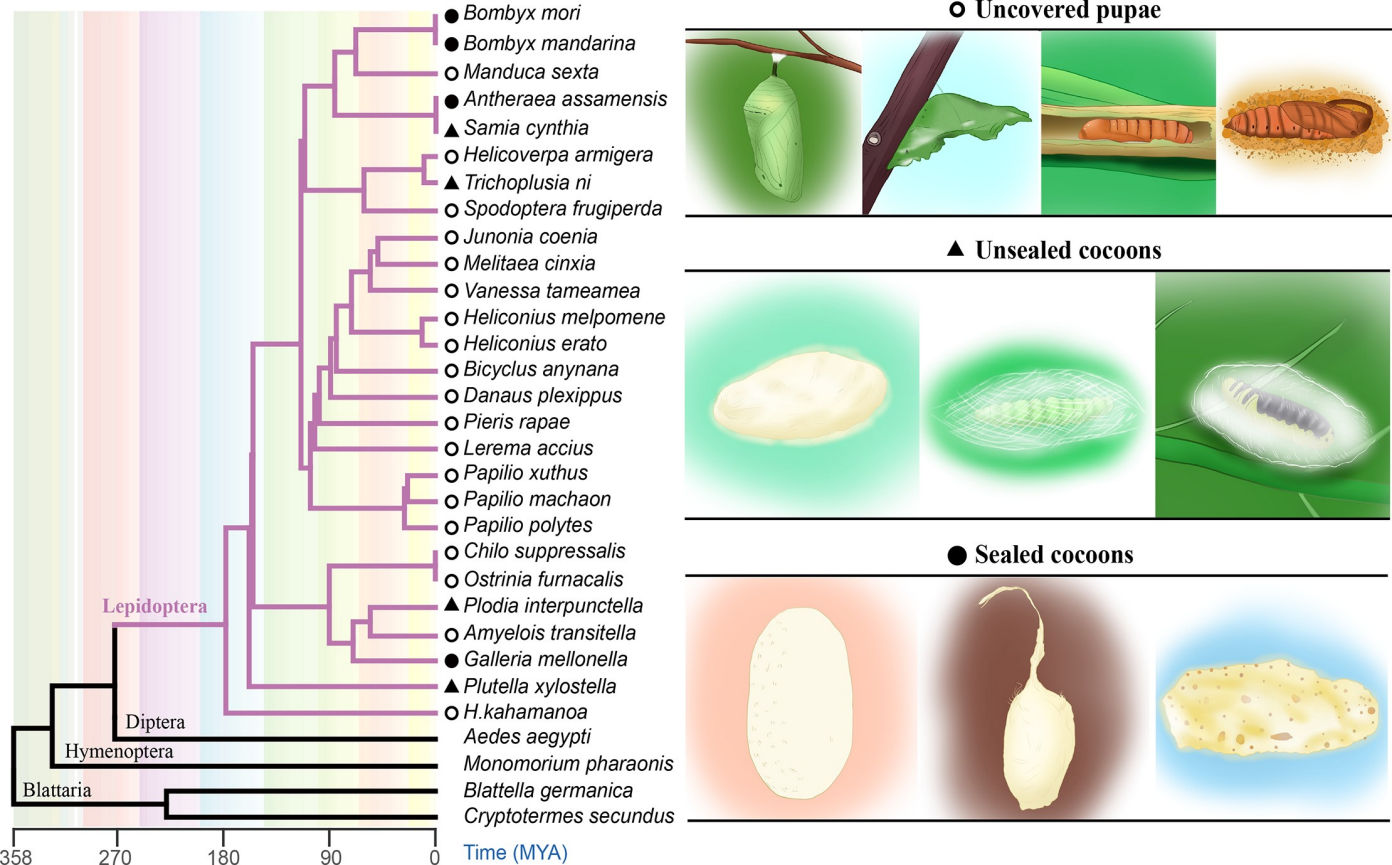
The diverse cocooning behaviors of lepidopteran insects. Based on the habits of cocooning or not and the characteristics of the cocoons, lepidopteran insects not spinning a cocoon, spinning a sealed cocoon, or having an unsealed cocoon were classified roughly and displayed on the right panels.

Although *cocoonase* is widely distributed in Lepidoptera, there are obvious differences in the cocooning behavior among them (Fig 3). Some butterflies do not spin a cocoon and the pupa is exposed to the environment, hanging upside down (pupa contigua) or leaning on branches (pupa adheraena), such as Nymphalidae and Papilionidae. Compared with the thick and sealed cocoons spun by the silkworm, *Bombyx mori* and *Galleria mellonella*, the cocoons of *Plutella xylostella* are extremely thin and transparent at both ends and there is a preformed eruption site in the cocoon of *Samia cynthia*. In order to detect the selection pressure on the *cocoonase* gene in these species displaying diverse cocooning behaviors, we used one-ratio and two-ratio models to analyze the selection pressure in each branch. The results of one-ratio model (model A in Table 1) analysis showed that the *cocoonase* gene in each branch is mainly affected by purifying selection during evolution (ω = 0.16308). We made also the hypothesis that the *cocoonase* gene in moths spinning sealed and unsealed cocoons were under different selection processes than in insects with uncovered pupae (model B in Table 1 and S2 Fig). The results showed no significant difference between the one-ratio and two-ratio models, but the moths spinning sealed cocoons were more strongly affected by purifying selection (ω1 = 0.13840, ω2 = 0.04881). Multiple sequence alignments also showed that the *cocoonase* genes are more conserved in insects spinning sealed cocoons compared with insects not spinning a cocoon or spinning unsealed cocoons (S3 Fig). Combining the diverse cocooning behaviors with the divergence time of Lepidoptera, the *cocoonase* gene appeared before the emergence of lepidopteran insects spinning cocoons, making the evolution of insects spinning cocoons possible. The strong purifying selection of *cocoonase* gene in moths spinning sealed cocoons revealed its essential role related to their decocooning process.

**Table 1:** Likelihood Ratio Tests of Selective Pressures on *cocoonase* in Lepidoptera.

Models	ω(*d*_*N*_*/d*_*s*_)	ln*L*	np	Models compared	*P* values
A. All branches share same ω	ω = 0.16308	-22225.452	123	A vs. B	0.06688
B. Moths and butterflies with uncovered pupae have ω1, moths spinning enclosed cocoons have ω2	ω0 = 0.16603 ω1 = 0.13840 ω2 = 0.04881	-22222.748	125		

ln*L*: Natural logarithm of the likelihood value; np: Number of parameters.

The one-ratio model (Model A) and two-ratio model (Model B) were used to estimate the selective pressures using Maximum-likelihood method. The ratio of non-synonymous to synonymous substitution rates-ω, was used to estimate the mean selection pressures on different branches of the phylogenetic tree.

### Knockout of *cocoonase* in the silkworm *Bombyx mori*

**Fig 4:**
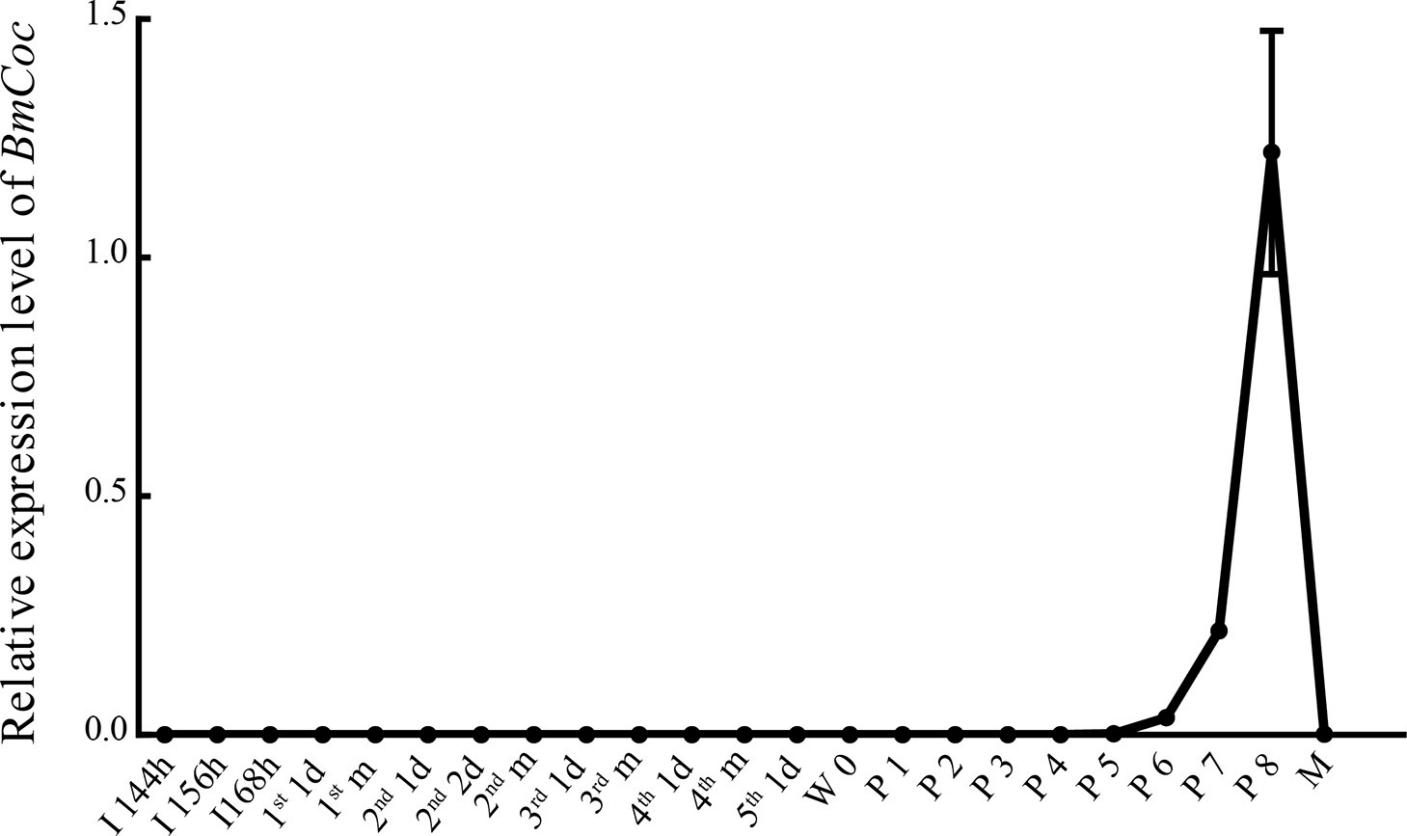
Temporal expression profile of *BmCoc* throughout the life cycle. Temporal expression profile of *BmCoc* was investigated throughout the life cycle from the stages of early embryo to the adult moth, and the silkworm ribosomal protein L3 (*RpL3*) was used as the internal reference. I144, I156, I168h: the time after incubation; 1^st^ 1d: day 1 of the first instar; 1^st^ m: the molting stage of the first instar; 2^nd^ 1d: day 1 of the second instar; 2^nd^ 2d: day 2 of the second instar; 2^nd^ m: the molting stage of the second instar; 3^rd^ 1d: day 1 of the third instar; 3^rd^ m: the molting stage of the third instar; 4^th^ 1d: day 1 of the fourth instar; 4^th^ m: the molting stage of the fourth instar; 5^th^ 1d: day 1 of the fifth instar; W0: the time the wandering stage was initiated; P1–P8: days 1–8 of pupae; M: moth.

In order to investigate the role of *cocoonase* in insects spinning cocoons, we knocked out *cocoonase* (*BmCoc*) using the CRISPR/Cas9 system in *Bombyx mori*. First, we examined the temporal expression profile of *BmCoc* through the entire life cycle to uncover the internal relationships between *BmCoc* gene expression and development. The results show that *BmCoc* is specifically expressed in the middle and late pupal stages, being initiated on the 5th day and gradually increasing to a peak on the 8th day and then decreasing to the end of the pupal stage (Fig 4). Previous studies have confirmed that cocoonase is synthesized in the maxillae, salivary glands and midgut [27–30], combined with its temporal specific expression pattern, we considered that knocking out *BmCoc* would not adversely affecting the growth and development of silkworm larva.

**Fig 5:**
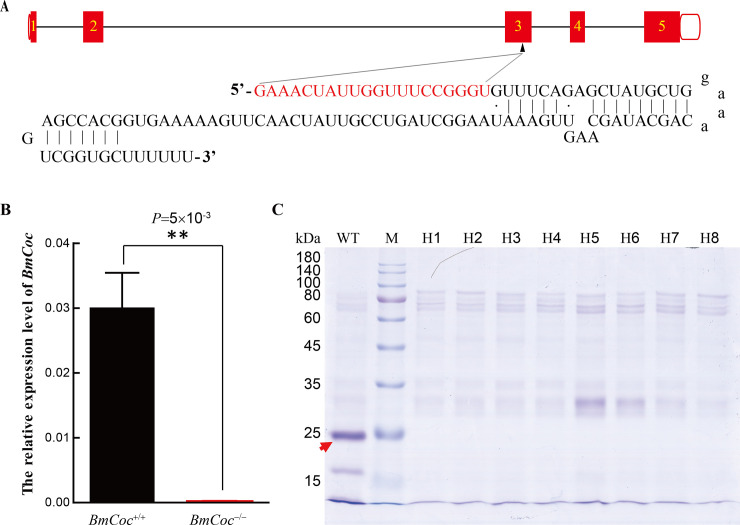
Knockout of *BmCoc* in silkworm by CRISPR/Cas9 system. (A) Gene structure with gRNA targeting exon 3 of *BmCoc*. (B) qRT-PCR analysis of the relative expression level of *BmCoc* in the wildtype (*BmCoc*^+/+^) and homozygous individuals (*BmCoc*^-/-^) on day 8 of the pupal stage, using cDNAs of heads as templates. The expression level was compared between *BmCoc*^-/-^ and *BmCoc*^+/+^ by two-tailed Student’s *t*-tests. ** *P* < 0.01. (C) The SDS-PAGE analysis for the proteins contained in the silkmoth-vomiting fluid collected from wild type and *BmCoc*^-/-^ individuals. The gel was stained with Coomassie brilliant blue R-250. The protein band indicated by the red arrow is the mature cocoonase with a predicted molecular weight of 23.7 kDa. M indicates a protein marker and H1-H8 lanes represent different fluid samples from eight homozygous individuals.

We thus designed a binary transgenic CRISPR/Cas9 system to knock out *BmCoc*, in which Cas9 and gRNA targeting *BmCoc* (Fig 5A) were expressed in two independent transgenic lines. After a series of hybridization, the homozygous individuals (*BmCoc*^-/-^) with 1 bp and 7 bp deletions were identified by fluorescence screening and mutation sequence analysis (S4 Fig), and the 7 bp-deletion line was used for subsequent experiments. The details of transgenic lines constructing and homozygotes screening are in the Methods and S4 Fig. The expression level of *BmCoc* was investigated at the mRNA and protein levels in *BmCoc*^-/-^ homozygotes. The mRNA level of *BmCoc* was extremely reduced compared to that in wild type individuals (Fig 5B). Meanwhile, samples of the silkmoth-vomiting fluid from wild type and *BmCoc*^-/-^ moths were collected and SDS-PAGE performed. Consistent with the results of qRT-PCR, there was no detectable mature cocoonase (predicted to be 23.7 kDa) in the samples of *BmCoc*^-/-^ silkworms, confirming the complete disruption of *BmCoc* (Fig 5C).

### *Cocoonase* is the determining factor allowing silk moths to escape from the cocoon

**Fig 6:**
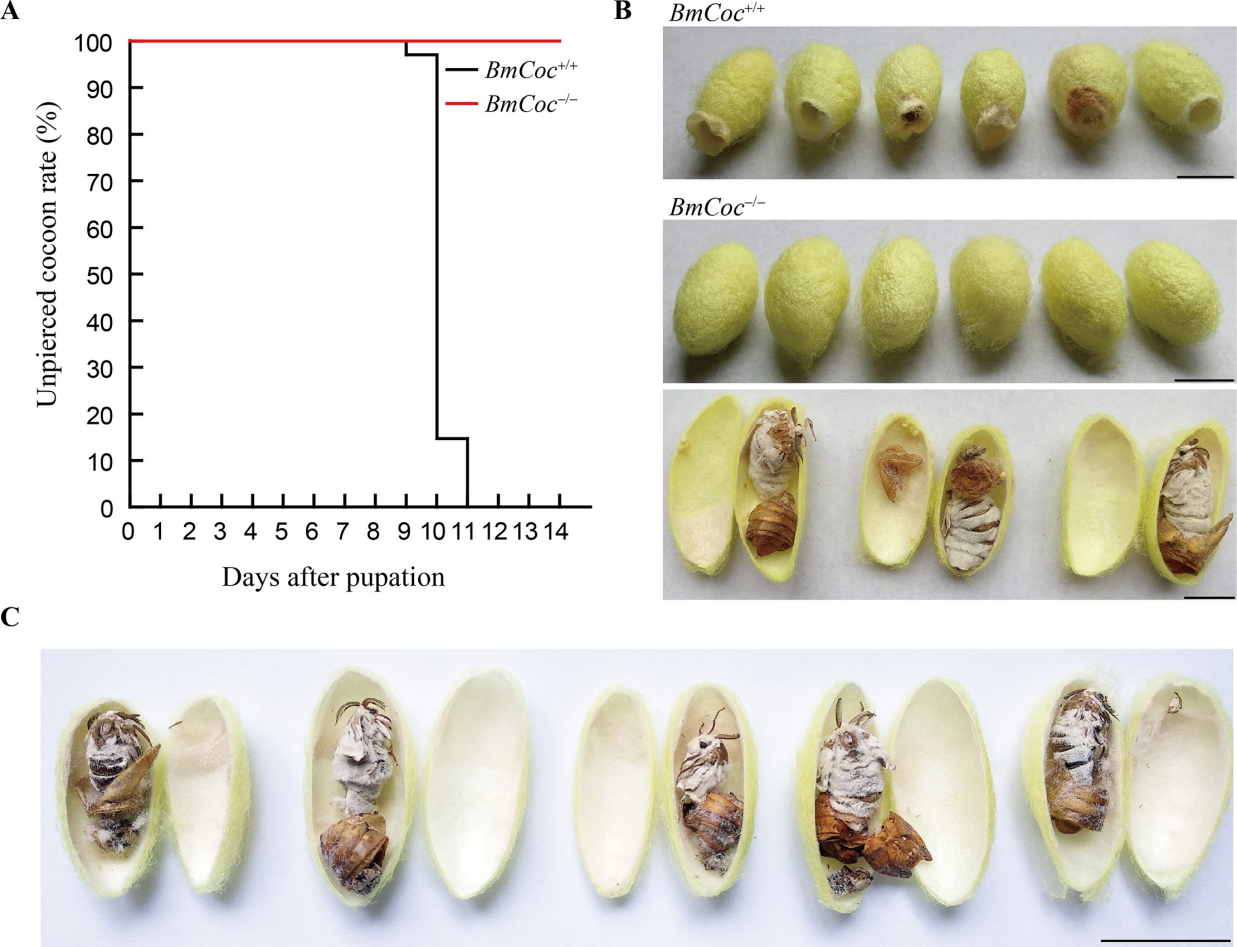
Effects of knocking out *BmCoc* on moth eclosion and emergence. (A) Unpierced cocoon rate of *BmCoc*^+/+^ and *BmCoc*^-/-^ individuals. One hundred individuals of wild type and *BmCoc*^-/-^ homozygotes were randomly selected for analysis. (B) Morphological observations of *BmCoc*^+/+^ and *BmCoc*^-/-^ cocoons. *BmCoc*^+/+^ cocoons with pierced holes caused by the moth escaping from the cocoon are shown on the top image. Intact cocoons of *BmCoc*^-/-^ and split cocoons with a successfully metamorphosed moth inside are shown below. Bar = 1 cm. (C) Morphological observations of homozygotes naturally stored for more than six months. Inside the cocoons are dried moths that have died naturally. Bar = 2 cm.

During the rearing process, we observed that the *BmCoc*^-/-^ homozygotes are able to grow, spin and cocoon normally. Eleven days after pupation, all the wild type moths exited from their cocoons, leaving pierced cocoon shells (Fig 6A). Meanwhile, the chimeras and heterozygotes of the *cocoonase* gene knockout mutation were also able to escape from the cocoon. However, all the *BmCoc*^-/-^ homozygotes could not escape from the cocoon, no matter whether the cocoon layer was thick or thin (Fig 6A and 6B). After splitting the cocoons, successfully metamorphosed adult moths could be observed, indicating no adverse impact on moth eclosion after disruption of *BmCoc* (Fig 6B). Moreover, there was no escapers in the offspring of the homozygous mutants. Six months later, we observed that the cocoons were as intact as ever, and the moths had died naturally without putrefaction (Fig 6C). Although there were detectable extra proteins in the silkmoth-vomiting fluid (Fig 5C), the moth stayed trapped in the cocoon due to the loss of cocoonase, which confirmed the determining role of *BmCoc* in facilitating moth escape from cocoon. Although we lack evidence from other species, the strong purifying selection on *cocoonase* suggests that *cocoonase* must play an indispensable role in assisting moth emergence in other lepidopteran insects spinning sealed cocoons.

## Discussion

The holometabolous insects undergo a particular metamorphosis stage called the pupa, which is the transitional stage from larva transforming into adult with a dramatic reorganization of tissues and organs. Spinning and cocooning protect pupae from unfavorable environments and predators [6–9]. The enzymatic mechanism allowing the escape of silk moths from their cocoons was reported [20–22], but it is still unclear whether there are other cofactors involved in the process and how widespread and pervasive is the cocoonase mechanism used for the emergence among insects.

Our investigation and analysis reveal that the *cocoonase* gene is specific to Lepidoptera. In the synteny analyses, *Hyposmocoma kahamanoa*, a kind of amphibious insect belonging to the genus *Hyposmocoma* (Cosmopterigidae) [40], was located at the base of the Lepidoptera lineage in the evolutionary tree of species. Although it does not construct cocoon, this species also has the *cocoonase* gene. Combined with the differentiation time of species and the cocooning behaviors of lepidopterans, it seemed that the *cocoonase* gene existed before the emergence of Lepidoptera insects spinning cocoons, which ensured that the adults could escape from their cocoons. With the increasing number of sequenced species, adding more basal lepidopteran insects is needed to strengthen the argument. In addition, insects spinning cocoons are distributed in multiple families that are not closely related, indicating that the cocooning behavior may have evolved convergently. Despite differences in cocooning behavior, the *cocoonase* gene is mainly affected by purifying selection in Lepidoptera and the selection is more intense in insects spinning sealed cocoons. The protease activities of *Bombyx mori* and *Antheraea Pernyi* were characterized by purified cocoonase through substrate degradation experiment [25, 32]. The results implied that the two enzymes have similar activities. In addition, there are other lepidopteran adults such as *Dicranura vinula* (Notodontidae), *Saturnia carpini* (Saturniidae), *Limacodes testudo* (Limacodidae) and *Halias prasinana* (Noctuide) were able to secrete vomiting fluid that could soften the cocoon assisting moth emergence [18, 19]. Combined with our research, we speculated that other cocoonase enzymes should have the conserved activities in dissolving cocoon. Further, genetic manipulation of *cocoonase* in the silkworm *Bombyx mori* forcefully illustrated its decisive role in adults escaping from sealed cocoons in lepidopteran insects. The gland producing silk and the cocoon silk proteins are very different among species [13, 14] and insects constructing sealed cocoons among other orders may have an additional factor (or other factors) for assisting adults in the escape from the cocoon.

Cocoon drying is an indispensable processing step in traditional silk production that involves killing the pupae and preventing them from metamorphosing into moths, which would destroy and contaminate the cocoons. In the *cocoonase* knocked-out silkworm strain reported here, the adults stayed trapped in the cocoons after moth development and showed no abnormities in the reproduction, hatchability and other cocoon-related traits (S5 Fig and S6 Fig). The *cocoonase* free silkworm leaves the cocoon without rupture and allows for long storage without heating or freezing. Thus, used as a novel breeding material, this new strain, upon further evaluation and utilization, should bring significant innovation to the silk industry. The identification of other key factors in the decocooning process in other orders will expand the scope of pest control. Indeed, the knockout of the *cocoonase* gene keeps the adult permanently trapped in the cocoon, failing to complete the generation cycle. Therefore, either inhibition or inactivation of the enzymatic action of the cocoonase or gene drive strategies in lepidopteran pests spinning sealed cocoons could control the pest population.

## Materials and methods

### The identification of cocoonase homologs

The cocoonase homologs from other species were obtained using BlastP [41, 42] on the NCBI (http://www.ncbi.nlm.nih.gov/), the lepbase (http://lepbase.org/) and the InsectBase (http://www.insect-genome.com/) websites, with the silkworm *cocoonase* protein sequence (NP_001103386.1) used as query object. After removing redundancy, the 295 sequences with a percent identity greater than 40% and *e-value*<1e-10 were aligned using the program Muscle built-in MEGA7 [43]; the gaps were deleted manually. The evolutionary history was inferred using the Maximum Likelihood method based on the Poisson correction model [44] with 1000 bootstrap replicates [45]. For better visualization and interpretation, the tree file was further edited in FigTree v1.4.3 and Adobe Illustrator CC 2018.

### Synteny analyses

Gene synteny analysis was conducted among the insects with cocoonase orthologs. The annotated genes within the 300 kb genomic region upstream and 500 kb genomic region downstream of *cocoonase* in NCBI and Lepbase were verified by Genome Browser. For un-annotated genes, the predicted protein sequences were annotated by blasting against NR database. Genes of *Samia cynthia* and *Antheraea assamensis* were predicted through FGENESH Program based on their genomic sequences.

### Phylogenetic analysis

For phylogenetic analysis of *cocoonase* genes among the species, the coding sequences were aligned through MUSCLE (Align Codons) and edited manually. Next, the phylogenetic tree was constructed using the Maximum Likelihood method based on the Kimura 2-parameter model [46] with 1000 bootstrap replicates. The sequence alignments and tree files were imported into EasyCodeML [47] for estimating the mean pressures on the different branches based on the ratio of non-synonymous to synonymous substitution rates (ω = *d*_N_/*d*_S_). Two branch models were selected for estimating the selection pressures. One-ratio model assumed that *cocoonase* in each branch shared the same ω value, while in the two-ratio model, we compared ω values on the foreground branches (moths with sealed cocoon and moths with unsealed cocoons) with the background branches (moths and butterflies with uncovered pupae).

### Silkworm strains and rearing

The multivoltine silkworm strain Dazao obtained from the Silkworm Gene Bank of Southwest University (Chongqing, China) was used for germ line transformation and subsequent experiments. Larvae were reared on fresh mulberry leaves at 25°C.

### RNA isolation and cDNA synthesis

Total RNA was isolated from the heads of individuals from the stages of early embryo to the adult moth using an Ultra-pure total RNA rapid extraction kit (BioTeke Corporation, CN) according to the manufacturer’s instructions. One microgram of total RNA was used to synthesize cDNA, using the PrimeScript RT reagent Kit with gDNA Eraser (Takara Biomedical Technology, Beijing, CN).

### Quantitative real-time PCR (qRT-PCR) analysis

Quantitative real-time PCR was performed to analyze the expression profiles of *BmCoc* (GenBank accession number: EF428980.1) from the stages of early embryo to the adult moth using individual cDNAs as templates. Quantitative mRNA measurements were performed using the Bio-Rad CFX96 sequence detection system with iTaq universal SYBR Green (Bio-Rad Laboratories, China), and the silkworm ribosomal protein L3 (*RpL3*, GenBank: AY769270.1) was used as the internal reference. The reactions were performed as follows: initial incubation at 95°C for 30 s followed by 40 cycles of 95°C for 5 s and 60°C for 30 s. The primers (DL-co-F/R and DL-rpl3-F/R) used in qRT-PCR to investigate the mRNA expression levels of *BmCoc* are provided in S1 Table.

### Construction of Cas9 and gRNA expression vectors

The type II CRISPR system from *Streptococcus pyogenes*, Known as CRISPR/Cas9 system, was used to knock out *cocoonase* (*BmCoc*) gene in silkworm, *Bombyx mori*. The primary Cas9 expression vector pCS7-Cas9 with nuclear localization sequence NLS and SV40 polyA was generously donated by ViewSolid Biotech. Company. The original promoter pCMV was replaced by the promoter of BmNPV immediate early protein 1 (IE1) in order to ensure that Cas9 could be expressed in the silkworm.

Twenty-nucleotide guiding RNA sequence complementary to the target with a protospacer-adjacent motif (PAM) were required to Cas9 directing. The RNA polymerase III–dependent U6 promoter (BmU6) was chosen to initiate the transcription of guiding RNAs, which requires a G at the 5' end of the sequence to be transcribed [48]. Thus, the target site must match the form GN19NGG. The coding sequence of *BmCoc* gene, which has five exons, was used to design target sites according to the above rule via the CRISPRdirect website (http://crispr.dbcls.jp/). A target site with the lowest off-target probability was finally selected to mediate a double-strand break in the exon 3 (Fig 5A). BmU6 promoter and gRNA scaffold sequences were synthesized and cloned into PUC57 vectors with two *Bbs* I cloning sites between them forming a PUC57-BmU6gRNA blank plasmid. The target site added endonuclease residues (S1 Table) were synthesized and linked to the PUC57-BmU6gRNA vector digested by *Bbs* I endonuclease.

The expression cassettes of the Cas9 and gRNA were subcloned to piggyBac blank vectors with nervous system-specifically expressed EGFP and RED markers, respectively.

### Acquisition of binary transgenic system

To obtain non-diapaused eggs, silkworm eggs that had been sterilized by hydrochloric acid were incubated at 15°C. The larvae were reared on fresh mulberry leaves under standard conditions until they had metamorphosed to adult moths that oviposited non-diapaused eggs (G_0_). PiggyBac-based Cas9 and gRNA expressing vectors were mixed in a 1:1 ratio with the helper plasmid pHA3PIG and injected into the non-diapaused embryos within 2 h after oviposition. The G_1_ embryos with green fluorescence in the ocelli were screened to obtain a transgenic line of Cas9 using a fluorescent microscope (Olympus), while embryos with red fluorescence were marked as a transgenic line of gRNA.

### Homozygote screening strategy

Cas9 transgenic lines were crossed with gRNA lines to produce F_1_ offspring with mosaic mutations. For the convenience of selection, we screened individuals with only red fluorescence (and/or no fluorescence) from the offspring of Cas9 and gRNA hybrids for extracting genomic DNA of wings and performing a mutation sequence assay. Male and female individuals identified as heterozygous were crossed to obtain homozygous individuals.

### DNA extraction and mutagenesis assay

Genomic DNA was extracted from the wings dissected from moths with DNAiso reagent (Takara) according to the manufacturer’s instructions. Concurrently, moths with wings dissected off were properly labeled and kept in a low-temperature refrigerator for later mating.

Sequences spanning the target site were amplified with the primers co-geno-F/R (S1 Table). The amplified PCR products were directly sequenced or subcloned into pMD19-T vectors (Takara, China). Clones were randomly chosen for Sanger sequencing to ascertain the mutated sequences.

### SDS-polyacrylamide gel electrophoresis

The day before moth eclosion, pupae were inverted with the head downward in Eppendorf tubes with an opening at the bottom, and the tubes were then placed in sterilized EP tubes. The next day, silkmoth-vomiting fluid was collected in the EP tube. The proteins contained in the fluid were quantified using a BCA kit (Beyotime) and separated by 10% SDS/PAGE. After electrophoresis finished, the excess gel was removed, and the gel containing the MARK protein and the samples was placed in Coomassie brilliant blue R-250 staining solution for 1 hour at room temperature, then placed in decolorizing solution for 4-24h until the blue background was almost completely removed.

### Statistical analysis

All the experiments in this study were performed with at least three replicates. All the data are expressed as the mean ± SEM. The differences between groups were examined by two-tailed Student *t*-test.

## Supporting information

S1 Fig The number of *cocoonase* genes identified in Lepidoptera. The order of the species is based on the species tree displayed on the left panel and the number of *cocoonase* gene in each species was shown in the right histogram. Moths are marked in green and butterflies are marked in light purple.
(TIF)

S2 Fig Phylogenetic analysis of cocoonase genes. The phylogenetic tree were constructed for phylogenetic analysis using maximum likelihood method based on the alignment of cocoonase coding sequences. The maximum likelihood tree was inferred using Kimura 2-parameter model with 1,000 bootstrap replicates. The numbers at the nodes indicated bootstrapping values. Insects not spinning a cocoon, spinning a sealed cocoon or having an unsealed cocoon were marked with rings, black circles and black triangles, respectively.
(TIF)

S3 Fig Amino acid alignment of cocoonases among lepidopterans. The multiple alignment was produced using Muscle and amino acids were further analyzed within three groups, insects spinning sealed or unsealed cocoon and insects do not spin a cocoon. The aligned amino acids with more than 50% conservation among different insects were colored. The diverse colors indicated the different types of amino acids. And the functional sites relevant to serine protease are shown above the sequences. The arrow implied the signal cleavage site; blue asterisk marked the three catalytic active sites of serine protease (histidine, aspartic acid and serine); the purple asterisk represented the substrate binding sites; the letter C stood for the cysteine that forms the disulfide bond.
(TIF)

S4 Fig The binary transgenic CRISPR/Cas9 system-mediated mutation and homozygote screening. A) Homozygote screening strategy. The Cas9 and gRNA transgenic lines, i.e., the parents (P), were hybridized to obtain the F_1_ progeny. Individuals simultaneously expressing Cas9 and gRNA among the F_1_ generation were screened for self-crossing (or backcrossed with wild-type individuals) to obtain the F_2_. Heterozygotes were screened out from the F_2_ population by mutation sequence detection. In order to remove transposable elements, only individuals without fluorescent markers were used for hybrids to obtain the F_3_. Homozygous mutant individuals with the expected phenotype were screened from the F_3_ population. B) Bright-field and fluorescent images of the positive binary transgenic moths. The red fluorescent image of the moth in ommatea indicated the gRNA-transgenic line and the green fluorescence (EGFP) implied the Cas9-transgenic line. Bar, 2 mm. C) Sanger sequencing identified a variety of mutant sequences in the hybrid progeny of the Cas9-transgenic line and the gRNA-transgenic line (F_1_). The mutated form is the deletion of sequences nearby the target site (RED marked), ranging from 14bp to 81bp. D) Polyacrylamide gel electrophoresis combined with Sanger sequencing identified the heterozygotes among F_2_ individuals and homozygotes among F_3_ individuals. The differential band marked with red numbers represents the sequence form of heterozygous mutants. The mutated sequence form of the heterozygous individuals was a 1 bp-deletion or a 7 bp-deletion shown with “-”.
(TIF)

S5 Fig The reproductive traits of *BmCoc*^-/-^ silkworms. The comparison of reproductive traits between wildtype (*BmCoc*^+/+^) and homozygous (*BmCoc*^-/-^) silkworms. A) The oviposition amount per female moth. The average oviposition numbers of *BmCoc*^+/+^ and *BmCoc*^-/-^ were 345.4 ±8 .044 and 351.8 ± 8.890, respectively. Both wildtype and homozygous were analyzed using twenty individuals. A two-tailed Student’s *t*-test showed no significant difference between the two groups. B) The hatching rates of *BmCoc*^+/+^ and *BmCoc*^-/-^ eggs. The hatching rates of *BmCoc*^+/+^ and *BmCoc*^-/-^ were 97.6% and 97.7%, respectively. The hatching rate was compared by a two-tailed Student’s *t*-test. n.s., not significant (*P*>0.05).
(TIF)

S6 Fig The key economic traits of cocoons between wild type and gene-edited silkworms. A) In wild type and *BmCoc*^-/-^ homozygous females, the cocoon weights were 1.174 ± 0.0106 g and 1.187 ± 0.0967 g; cocoon shell weights were 0.139 ± 0.0018 g and 0.138 ± 0.0022 g, and the cocoon shell rates were 11.85% ±0.10% and 11.60% ± 0.13%, respectively. B) In wild type and *BmCoc*^-/-^ homozygous males, cocoon weights were 0.873 ± 0.0059 g and 0.864 ± 0.0064 g; cocoon shell weights were 0.135 ± 0.0014 g and 0.132 ± 0.0014 g, and cocoon shell rates were 15.51% ± 0.11% and 15.25% ± 0.09%, respectively. Fifty cocoons were selected randomly to investigate cocoon weight, cocoon shell weight and cocoon shell rate. The changes of each group were compared with that of the wild type (*BmCoc*^+/+^) by two-tailed Student’s *t*-tests. n.s., not significant (*P*>0.05). In the box plot, center lines represent median values; box limits represent the interquartile range; whiskers extend 1.5 times the interquartile range, and dots represent outliers.
(TIF)

S1 Table Primers used in this study. (DOCX)

## References

[1] Stork NE. How many species of insects and other terrestrial arthropods are there on Earth?. Annu Rev Entomol. 2018; 63:31–45. 10.1146/annurev-ento-020117-043348 28938083

[2] Koštál V. Eco-physiological phases of insect diapause. J Insect Physiol. 2006; 52(2), 113–127. 10.1016/j.jinsphys.2005.09.008 16332347

[3] Ruxton GD, Sherratt TN, Speed MP. Avoiding attack: the evolutionary ecology of crypsis, warning signals and mimicry. Oxford University Press, Oxford, UK; 2004.

[4] Dingle H. Migration Strategies of Insects. Science. 1972; 175 (4028), 1327–1335. 10.1126/science.175.4028.1327 17813822

[5] Chapman JW, Reynolds DR, Wilson K. Long-range seasonal migration in insects: mechanisms, evolutionary drivers and ecological consequences. Ecol Lett. 2015; 18(3), 287–302. 10.1111/ele.12407 25611117

[6] Bartell DP, Sanborn JR, Wood KA. Insecticide Penetration of Cocoons Containing Diapausing and Nondiapausing Bathyplectes curculionis, an Endoparasite of the Alfalfa Weevil. Environmental Entomology. 1976; 5:659–61.

[7] Halpern M, Gasith A, Broza M. Does the tube of a benthic chironomid larva play a role in protecting its dweller against chemical toxicants? Hydrobiologia. 2002; 470:49–55.

[8] Stevens DJ, Hansell MH, Freel JA, Monaghan P. Developmental trade-offs in caddis flies: increased investment in larval defence alters adult resource allocation. Proc Biol Sci. 1999; 266(1423):1049.

[9] Danks HV. The roles of insect cocoons in cold conditions. European Journal of Entomology. 2004; 101(3):433–7.

[10] Jenkins MF. Cocoon building and the production of silk by the mature larva of *Dianous coerulescens Gyllenhal* (Coleoptera: Staphylinidae). Trans R entomol Soc London. 1958; 110:287–301.

[11] Trouvelot L. The American Silk Worm. Am Nat. 1867; 1:30–8.

[12] Donald LJ, Shaw MR, Takahashi M, Yanechin B. Cocoon silk chemistry of non-cyclostome Braconidae, with remarks on phylogenetic relationships within the Microgastrinae (*Hymenoptera*: Braconidae). Journal of Natural History. 2010; 38:2167–81.

[13] Rudall KM, Kenchington W. Arthropod Silks: The Problem of Fibrous Proteins in Animal Tissues. Annual Review of Entomology. 1971; 16:73–96.

[14] Sutherland TD, Young JH, Weisman S, Hayashi CY, Merritt DJ. Insect silk: one name, many materials. Annu Rev Entomol. 2010; 55:171–88. 10.1146/annurev-ento-112408-085401 19728833

[15] Ishii S, Inokuchi T, Kanazawa J, Tomizawa C. Studies on the cocoon of the oriental moth, *Monema* (*Cnidocampa*) *flavescens*, (lepidoptera: limacodidae). III. Structure and composition of the cocoon in relation to hardness. Japanese Journal of Applied Entomology and Zoology. 1984; 28(4), 269–273.

[16] Harcourt DG. Biology of the Diamondback Moth, *Plutella maculipennis* (Curt.) (*Lepidoptera*: Plutellidae), in Eastern Ontario. II. Life-History, Behaviour, and Host Relationships. The Canadian Entomologist. 1957; 89(12), 554–564.

[17] Yu R, Shi M, Huang F, & Chen X. Immature Development of *Cotesia vestalis* (*Hymenoptera*: Braconidae), an Endoparasitoid of *Plutella xylostella* (Lepidoptera: Plutellidae). Annals of the Entomological Society of America. 2008; 101(1), 189–196.

[18] Latter OH. XVIII. The secretion of potassium hydroxide by *Dicranura vinula* (imago), and the emergence of the imago from the cocoon. Transactions of the Royal Entomological Society of London. 2009; 40(4), 287–292.

[19] Latter OH. XIV. Further Notes on the Secretion of Potassium Hydroxide by *Dicranura vinula* (imago), and similar Phenomena in other Lepidoptera. Transactions of the Royal Entomological Society of London. 2009; 43(3), 399–409.

[20] Duspiva F. The enzymatic processes when the silk spinner (*Bombyx mori* L.) breaks through the cocoon shell. Journal of Natural Science B. 1950; 5b:273–81.

[21] Kafatos FC, Williams CM. Enzymatic Mechanism for the Escape of Certain Moths from Their Cocoons. Science. 1964; 146(3643):538–40. 10.1126/science.146.3643.538 17806809

[22] Kafatos FC, Tartakoff AM, Law JH. Cocoonase. I. Preliminary characterization of a proteolytic enzyme from silk moths. The Journal of Biological Chemistry. 1967; 242:1477–87. 6023217

[23] Wu Y, Wang W, Wang BLD and Shen W, Cloning and expression of the *cocoonase* gene from *Bombyx mori*. *Sci Agric Sin*. 2008; 41:3277–3285.

[24] Yamazaki Y, Ogawa K and Kanekatsu R, Isolation of cocoonase from the silkworm, *Bombyx mori*, by a high performance liquid chromatography and catalytic specificity. J Seric Sci Jpn. 1992; 61:228–235.

[25] Hidetoshi T, Keiji K and Mitsuhiro M, Proteolytic characterization of cocoonase from the domestic silk moth, Bombyx mori. Pept Sci 42:479–482 (2005).

[26] Fukumori H, Teshiba S, Shigeoka Y, Yamamoto K, Banno Y and Aso Y, Purification and characterization of cocoonase from the silkworm *Bombyx mori*. Biosci Biotechnol Biochem. 2014; 78:202–211. 10.1080/09168451.2014.878215 25036672

[27] Eguchi M, Iwamoto A. Proteases in the pupal midgut of the silkworm, *Bombyx mori* L. II. Hydrolysis of the solubilized fibroin and native silk. J Sericult Sci Japan. 1973; 42(2):144–50.

[28] Eguchi M, Iwamoto A. Rôle of the midgut, crop, and maxillae of *Bombyx mori* in the production of cocoon-digesting enzyme. J Insect Physiol. 1975; 21(7):1365–82.

[29] Wang H, Zhang C, Cui W, Liu X, Zhou Y, Cai Y, et al Studies on Secretory Organs of Cocoonase and Silkmoth-vomiting Fluid of Silkworm, *Bombyx mori*. Acta Sericologica Sinica. 2005; 31(2):136–41.

[30] Zhang C, Cui W, Guo Y, Wang Y, Mu Z. Ultrastructure changes and function of the midgut and salivary glands in *Bombyx mori* during the pupal-adult metamorphism. Acta Entomologica Sinica. 2007; 50(8):769–74.

[31] Prasad BC, Pandey JP and Sinha AK, Study of *Antheraea mylitta* cocoonase and its use in cocoon cooking. Am J Food Technol. 2012; 7:320–325.

[32] Geng P, Lin L, Li Y, Fan Q, Wang N, Song L, et al A novel fibrin(ogen)olytic trypsin-like protease from Chinese oak silkworm (*Antheraea pernyi*): purification and characterization. Biochem Biophys Res Commun. 2014; 445:64–67 10.1016/j.bbrc.2014.01.155 24491553

[33] Yang J, Wang W, Li B, Wu Y, Wu H and Shen W, Expression of cocoonase in silkworm (*Bombyx mori*) cells by using a recombinant baculovirus and its bioactivity assay. Int J Biol. 2009; 1:107–112.

[34] Rodbumrer P, Arthan D, Uyen U, Yuvaniyama J, Svasti J and Wongsaengchantra PY, Functional expression of a *Bombyx mori* cocoonase: potential application for silk degumming, Acta Biochim Biophys Sin. 2012; 44:974–983. 10.1093/abbs/gms090 23169343

[35] Unajak S., Aroonluke S., & Promboon A. An active recombinant cocoonase from the silkworm *Bombyx mori*: bleaching, degumming and sericin degrading activities. J Sci Food Agr. (2014); 95(6), 1179–1189. 25042939 10.1002/jsfa.6806

[36] Zhu Y, Wang Z, He N, Li C. Enzymatic Activity and Protein Species Identification of Spit Liquid from *Bombyx mori* Moths. Science of Sericulture. 2014; 40(03):0452–57.

[37] Harpel D, Cullen DA, Ott SR, Jiggins CD, Walters JR. Pollen feeding proteomics: Salivary proteins of the passion flower butterfly, Heliconius melpomene. Insect Biochem Mol Biol. 2015;63:7–13. 10.1016/j.ibmb.2015.04.004 25958827

[38] Smith G, Macias-Munoz A, Briscoe AD. Gene Duplication and Gene Expression Changes Play a Role in the Evolution of Candidate Pollen Feeding Genes in Heliconius Butterflies. Genome Biol Evol. 2016;8(8):2581–96. 10.1093/gbe/evw180 27553646 PMC5010911

[39] Smith G, Kelly JE, Macias-Munoz A, Butts CT, Martin RW, Briscoe AD. Evolutionary and structural analyses uncover a role for solvent interactions in the diversification of cocoonases in butterflies. Proc Biol Sci. 2018; 285:2017–37. 10.1098/rspb.2017.2037 PMC5784194 29298934

[40] Rubinoff D, Schmitz P. Multiple aquatic invasions by an endemic, terrestrial Hawaiian moth radiation. Proceedings of the National Academy of Sciences. 2010; 107(13), 5903–5906. 10.1073/pnas.0912501107 PMC2851915 20308549

[41] Altschul SF, Madden TL, Schäffer AA, Zhang J, Zhang Z, Miller W, et al Gapped BLAST and PSI-BLAST: a new generation of protein database search programs. Nucleic Acids Research. 1997; 25(17):3389–402. 10.1093/nar/25.17.3389 9254694 PMC146917

[42] Altschul SF, Wootton JC, Gertz EM, Agarwala R, Morgulis A, Schaffer AA, et al Protein database searches using compositionally adjusted substitution matrices. FEBS J. 2005; 272(20):5101–9. 10.1111/j.1742-4658.2005.04945.x 16218944 PMC1343503

[43] Kumar S, Stecher G, Tamura K. MEGA7: Molecular Evolutionary Genetics Analysis Version 7.0 for Bigger Datasets. Mol Biol Evol. 2016; 33(7):1870–1874. 10.1093/molbev/msw054 27004904 PMC8210823

[44] Zuckerkandl E and Pauling L. Evolutionary divergence and convergence in proteins. In: Bryson V and Vogel HJ, editors. Evolving Genes and Proteins; 1965. pp. 97–166.

[45] Felsenstein J. Confidence limits on phylogenies: An approach using the bootstrap. Evolution. 1985; 39(4):783–91. 10.1111/j.1558-5646.1985.tb00420.x 28561359

[46] Kimura M. A Simple Method for Estimating Evolutionary Rates of Base Substitutions Through Comparative Studies of Nucleotide Sequences. J Mol Evol. 1980; (16):111–20. 7463489 10.1007/BF01731581

[47] Gao F, Chen C, Arab DA, Du Z, He Y, Ho SYW. EasyCodeML: A visual tool for analysis of selection using CodeML. Ecol Evol. 2019; 9(7):3891–8. 10.1002/ece3.5015 31015974 PMC6467853

[48] Sander J. D., & Joung J. K. CRISPR-Cas systems for editing, regulating and targeting genomes. NAT BIOTECHNOL. 2014; 32(4), 347–355. 10.1038/nbt.2842 24584096 PMC4022601

