# Supplementary figures and images for: Cocoonase is indispensable for Lepidoptera insects breaking the sealed cocoon
Source: PLoS Genet. 2020 Sep 28;16(9):e1009004. doi: 10.1371/journal.pgen.1009004 (PMC7544147; doi:10.1371/journal.pgen.1009004)

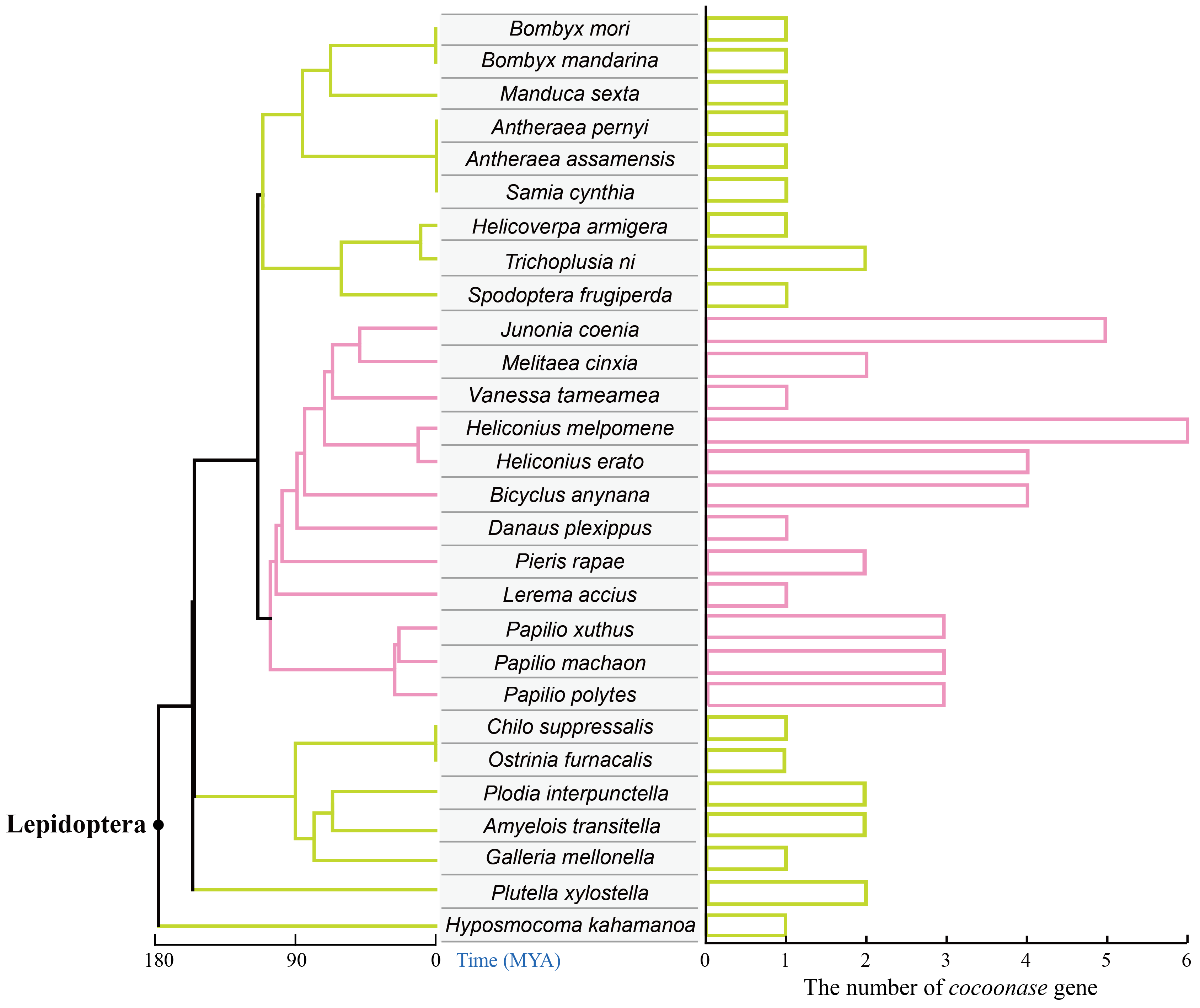

Supplement: S1 Fig — The order of the species is based on the species tree displayed on the left panel and the number of cocoonase gene in each species was shown in the right histogram. Moths are marked in green and butterflies are marked in light purple. (TIF) [file pgen.1009004.s001.tif]

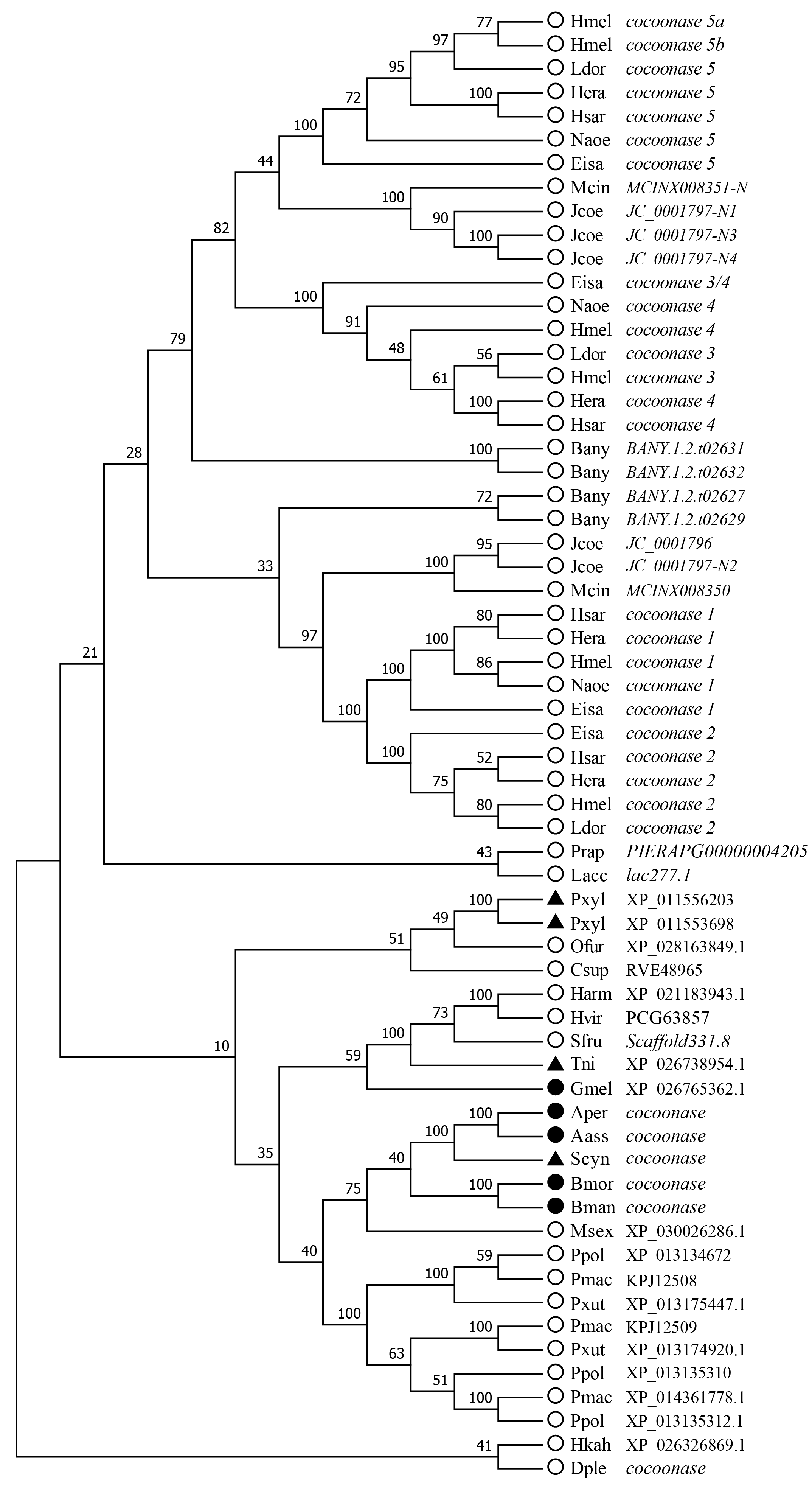

Supplement: S2 Fig — The phylogenetic tree were constructed for phylogenetic analysis using maximum likelihood method based on the alignment of cocoonase coding sequences. The maximum likelihood tree was inferred using Kimura 2-parameter model with 1,000 bootstrap replicates. The numbers at the nodes indicated bootstrapping values. Insects not spinning a cocoon, spinning a sealed cocoon or having an unsealed cocoon were marked with rings, black circles and black triangles, respectively. (TIF) [file pgen.1009004.s002.tif]

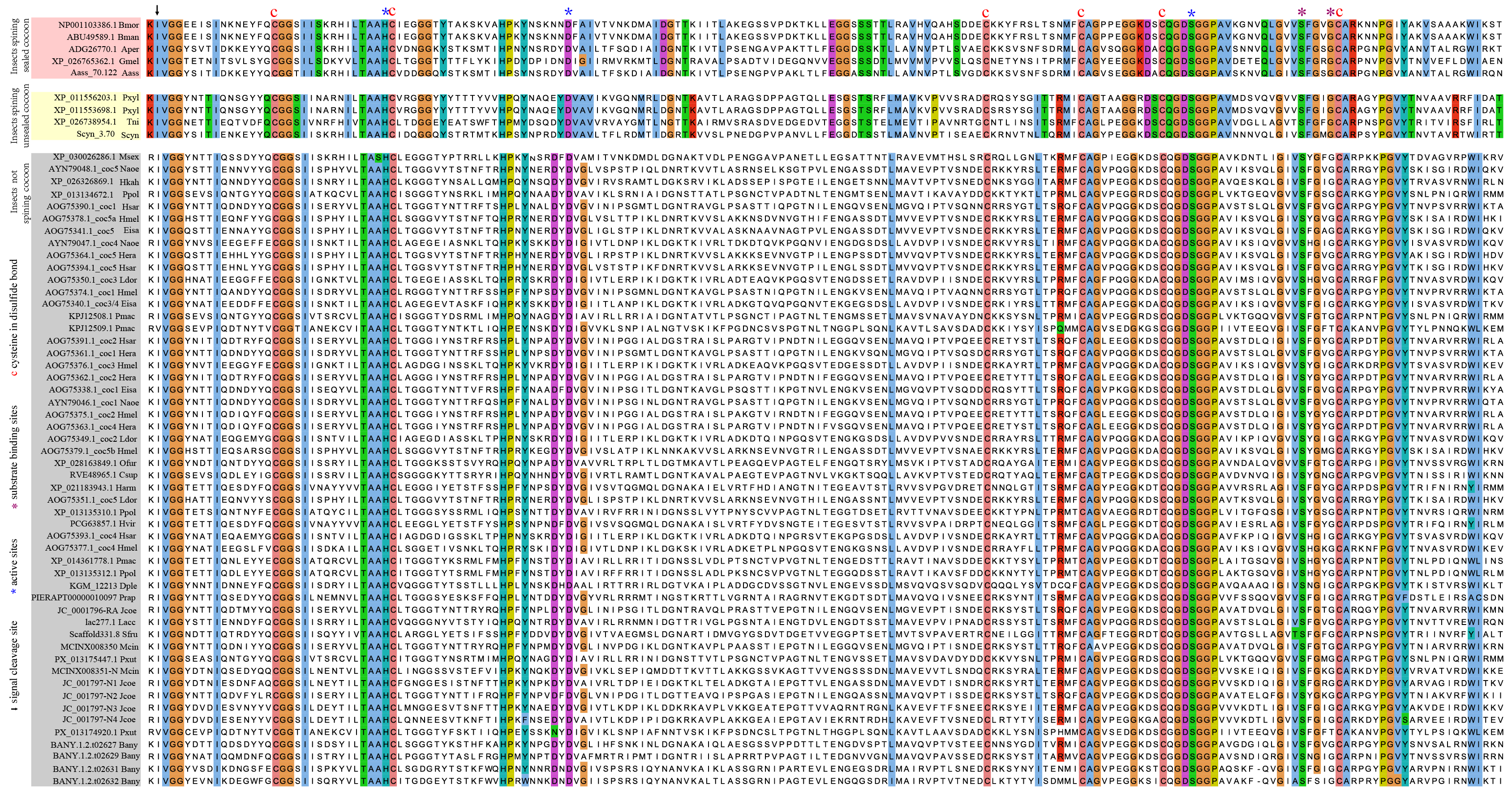

Supplement: S3 Fig — The multiple alignment was produced using Muscle and amino acids were further analyzed within three groups, insects spinning sealed or unsealed cocoon and insects do not spin a cocoon. The aligned amino acids with more than 50% conservation among different insects were colored. The diverse colors indicated the different types of amino acids. And the functional sites relevant to serine protease are shown above the sequences. The arrow implied the signal cleavage site; blue asterisk marked the three catalytic active sites of serine protease (histidine, aspartic acid and serine); the purple asterisk represented the substrate binding sites; the letter C stood for the cysteine that forms the disulfide bond. (TIF) [file pgen.1009004.s003.tif]

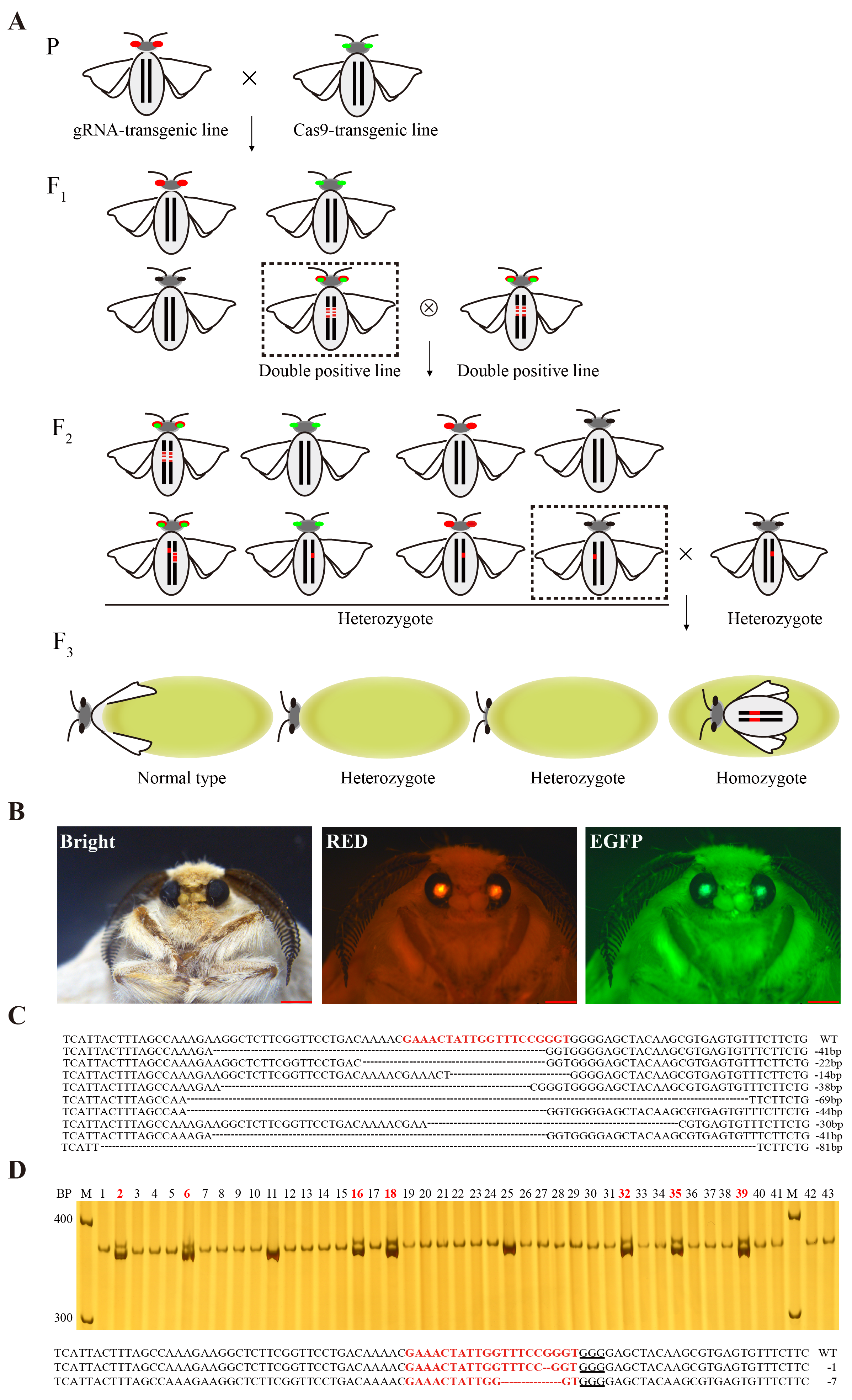

Supplement: S4 Fig — A) Homozygote screening strategy. The Cas9 and gRNA transgenic lines, i.e., the parents (P), were hybridized to obtain the F1 progeny. Individuals simultaneously expressing Cas9 and gRNA among the F1 generation were screened for self-crossing (or backcrossed with wild-type individuals) to obtain the F2. Heterozygotes were screened out from the F2 population by mutation sequence detection. In order to remove transposable elements, only individuals without fluorescent markers were used for hybrids to obtain the F3. Homozygous mutant individuals with the expected phenotype were screened from the F3 population. B) Bright-field and fluorescent images of the positive binary transgenic moths. The red fluorescent image of the moth in ommatea indicated the gRNA-transgenic line and the green fluorescence (EGFP) implied the Cas9-transgenic line. Bar, 2 mm. C) Sanger sequencing identified a variety of mutant sequences in the hybrid progeny of the Cas9-transgenic line and the gRNA-transgenic line (F1). The mutated form is the deletion of sequences nearby the target site (RED marked), ranging from 14bp to 81bp. D) Polyacrylamide gel electrophoresis combined with Sanger sequencing identified the heterozygotes among F2 individuals and homozygotes among F3 individuals. The differential band marked with red numbers represents the sequence form of heterozygous mutants. The mutated sequence form of the heterozygous individuals was a 1 bp-deletion or a 7 bp-deletion shown with “-”. (TIF) [file pgen.1009004.s004.tif]

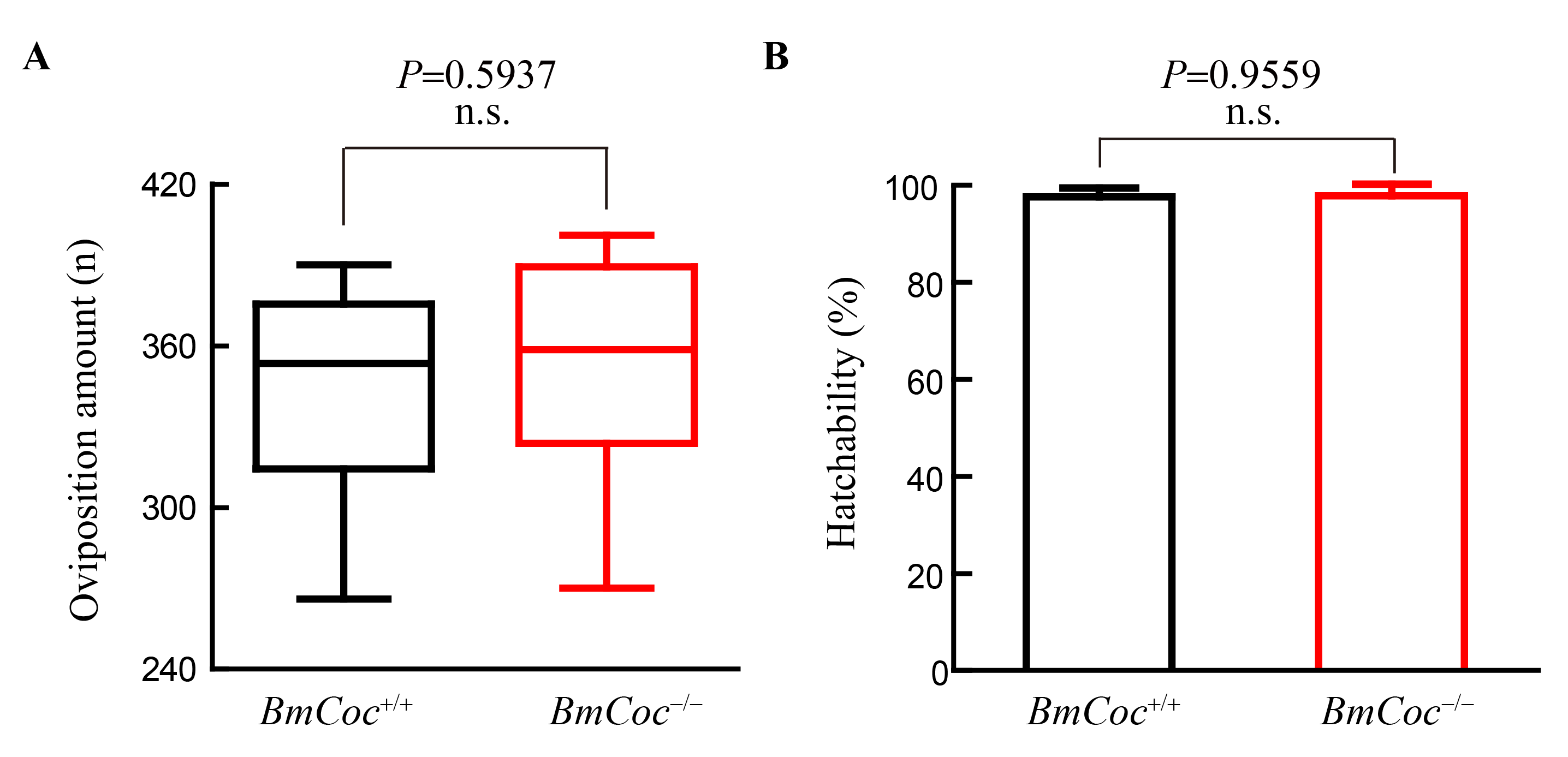

Supplement: S5 Fig — The comparison of reproductive traits between wildtype (BmCoc+/+) and homozygous (BmCoc-/-) silkworms. A) The oviposition amount per female moth. The average oviposition numbers of BmCoc+/+ and BmCoc-/- were 345.4 ±8 .044 and 351.8 ± 8.890, respectively. Both wildtype and homozygous were analyzed using twenty individuals. A two-tailed Student’s t-test showed no significant difference between the two groups. B) The hatching rates of BmCoc+/+ and BmCoc-/- eggs. The hatching rates of BmCoc+/+ and BmCoc-/- were 97.6% and 97.7%, respectively. The hatching rate was compared by a two-tailed Student’s t-test. n.s., not significant (P>0.05). (TIF) [file pgen.1009004.s005.tif]

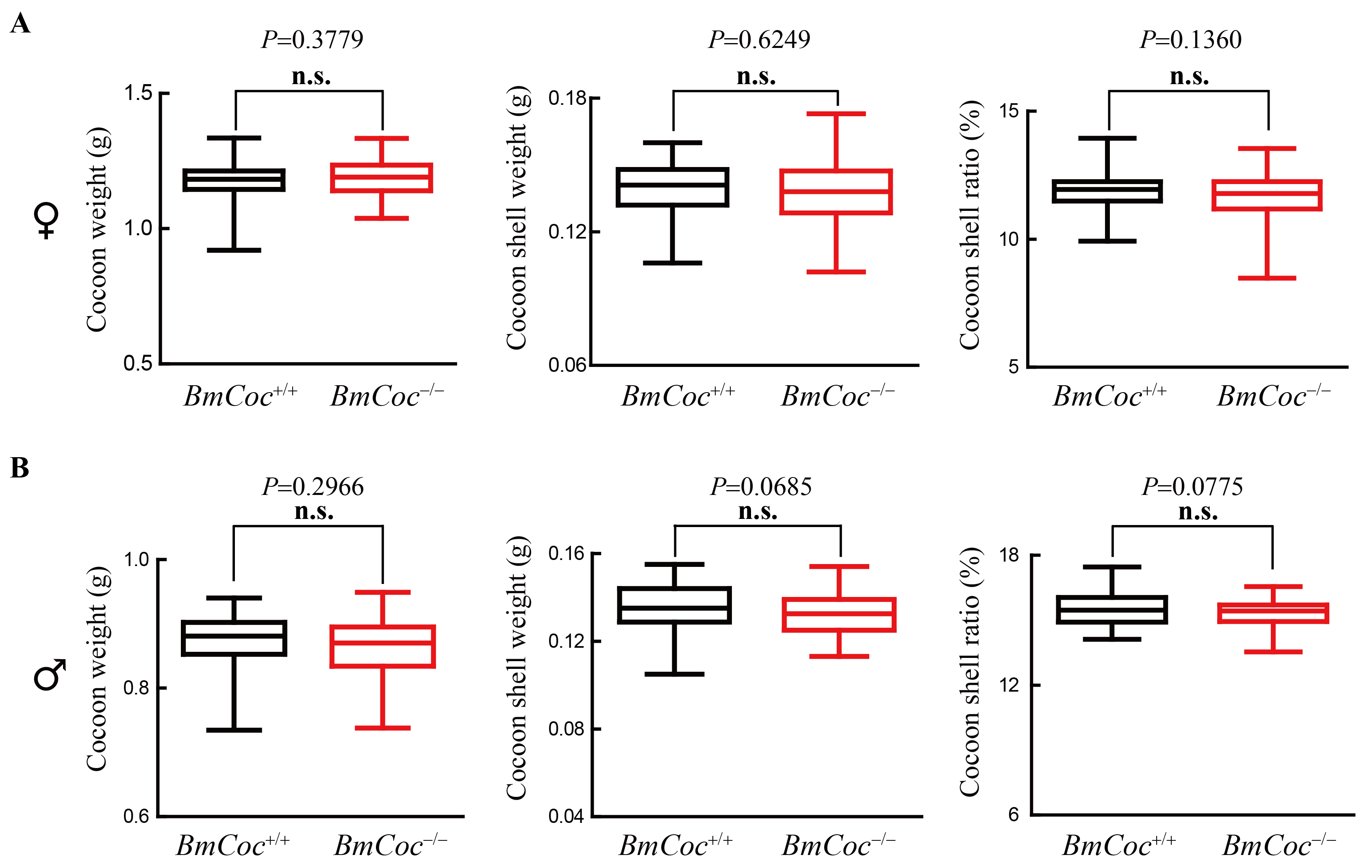

Supplement: S6 Fig — A) In wild type and BmCoc-/- homozygous females, the cocoon weights were 1.174 ± 0.0106 g and 1.187 ± 0.0967 g; cocoon shell weights were 0.139 ± 0.0018 g and 0.138 ± 0.0022 g, and the cocoon shell rates were 11.85% ±0.10% and 11.60% ± 0.13%, respectively. B) In wild type and BmCoc-/- homozygous males, cocoon weights were 0.873 ± 0.0059 g and 0.864 ± 0.0064 g; cocoon shell weights were 0.135 ± 0.0014 g and 0.132 ± 0.0014 g, and cocoon shell rates were 15.51% ± 0.11% and 15.25% ± 0.09%, respectively. Fifty cocoons were selected randomly to investigate cocoon weight, cocoon shell weight and cocoon shell rate. The changes of each group were compared with that of the wild type (BmCoc+/+) by two-tailed Student’s t-tests. n.s., not significant (P>0.05). In the box plot, center lines represent median values; box limits represent the interquartile range; whiskers extend 1.5 times the interquartile range, and dots represent outliers. (TIF) [file pgen.1009004.s006.tif]
