# Supplementary material for: Cocoonase is indispensable for Lepidoptera insects breaking the sealed cocoon
Source: PLoS Genet. 2020 Sep 28;16(9):e1009004. doi: 10.1371/journal.pgen.1009004 (PMC7544147; doi:10.1371/journal.pgen.1009004)
Supplement: S1 Table — (DOCX) [file pgen.1009004.s007.docx]

**S1 Table.** Primers used in this study

| Primer name | Primer sequence(5’-3’) | Primer purpose |
| --- | --- | --- |
| IE1-F | *GCGCGCCGGC*ATCAATGTCTTTGTGATGCGC | Amplification of IE1 promoter |
| IE1-R | *CGGGGTACC*TTGCGTCATAGTCGTTTGGTT | Amplification of IE1 promoter |
| co-sg-F | *AAGT*GAAACTATTGGTTTCCGGGT | Construction of sgRNA expression vector |
| co-sg-R | *AAAC*ACCCGGAAACCAATAGTTTC | Construction of sgRNA expression vector |
| co-geno-F | GTGGCTCATCCGAAATACAA | Identification of mutagenesis |
| co-geno-R | CAATGTCTAGCTGCTGGATCTT | Identification of mutagenesis |
| DL-co-F | GGTGGCTCATCCGAAATACAAT | qRT-PCR |
| DL-co-R | TTCTCGACCTTATTACGTTCGAAG | qRT-PCR |
| DL-rpl3-F  DL-rpl3-R | CGGTGTTGTTGGATACATTGAG  GCTCATCCTGCCATTTCTTACT | qRT-PCR  qRT-PCR |

Note: Italics indicate the restriction endonuclease site and protecting base or restriction enzyme residue.
